# EMMPRIN Promotes Melanoma Cells Malignant Properties through a HIF-2alpha Mediated Up-Regulation of VEGF-Receptor-2

**DOI:** 10.1371/journal.pone.0012265

**Published:** 2010-08-31

**Authors:** Faten Bougatef, Suzanne Menashi, Farah Khayati, Benyoussef Naïmi, Raphaël Porcher, Marie-Pierre Podgorniak, Guy Millot, Anne Janin, Fabien Calvo, Céleste Lebbé, Samia Mourah

**Affiliations:** 1 Inserm, UMR-S 940 Laboratoire de Pharmacologie, Paris, France; 2 Université Paris 7- Denis Diderot, Paris, France; 3 AP-HP, Hôpital Saint-Louis, Paris, France; 4 CNRS-UMR 7149, Laboratoire CRRET, Créteil, France; 5 Université Paris12–94000, Créteil, France; 6 Inserm, U717, Department of Biostatistics and Medical Data Processing, Paris, France; 7 Inserm, U728, Paris, France; 8 Department of Dermatology Hôpital Saint Louis, Paris, France; Institut Pasteur, France

## Abstract

EMMPRIN's expression in melanoma tissue was reported to be predictive of poor prognosis. Here we demonstrate that EMMPRIN up-regulated VEGF receptor-2 (VEGFR-2) in two different primary melanoma cell lines and consequently increased migration and proliferation of these cells while inhibiting their apoptosis. SiRNA inhibition of VEGFR-2 expression abrogated these EMMPRIN effects. EMMPRIN regulation of VEGFR-2 was mediated through the over-expression of HIF-2α and its translocation to the nucleus where it forms heterodimers with HIF-1β. These results were supported by an *in vivo* correlation between the expression of EMMPRIN with that of VEGFR-2 in human melanoma tissues as well as with the extent of HIF-2α localization in the nucleus. They demonstrate a novel mechanism by which EMMPRIN promotes tumor progression through HIF-2α/VEGFR-2 mediated mechanism, with an autocrine role in melanoma cell malignancy. The inhibition of EMMPRIN in cancer may thus simultaneously target both the VEGFR-2/VEGF system and the matrix degrading proteases to block tumor cell growth and invasion.

## Introduction

Malignant melanoma is capable of rapid progression and the prognosis of the advanced stages of the disease is extremely poor. Angiogenesis represents an essential step in its multistage progression and antiangiogenic agents are currently tested in patients with advanced melanoma [1]. A vital role in tumor angiogenesis is played by the Vascular Endothelial Growth Factor (VEGF), but the associated expression of both VEGF and its receptors by most advanced stage melanomas also suggests the possibility of an autocrine loop within the melanoma cells. This is supported by the demonstrated ability of VEGF to stimulate proliferation and migration of these cells [2]. Although two VEGF receptors are known, VEGF-R1/flt-1 and VEGFR-2/KDR/flk-1, VEGF was shown to signal mainly through VEGFR-2, which upon ligand binding becomes tyrosine phosphorylated and activates multiple signaling networks [3]. In accordance with this, patients with high VEGFR-2 expression in melanoma lesions were shown to be more likely to respond to Sorafenib therapy, a multi-target kinase inhibitor [4]. This is likely due to inhibition of both angiogenesis and cell proliferation driven by the presence of a VEGF/VEGFR-2 autocrine loop in tumor cells.

Extracellular MMP inducer (EMMPRIN/CD147), a membrane glycoprotein greatly enriched on the surface of tumor cells is known to stimulate tumor and neighbouring stromal cells, such as fibroblasts and endothelial cells, to increase their synthesis of several MMPs [5], [6], [7], [8], [9]. We have shown that EMMPRIN is not only an MMP inducer but can also increase the urokinase plasminogen activating system in tumor and endothelial cells (ECs), further increasing its proteolytic potential [10]. EMMPRIN is also implicated in lactate efflux, essential for tumor cell invasion, via its cotransporter MCT4 [11]. Indeed, the conversion of glucose to lactic acid in the presence of oxygen, generally known as aerobic glycolysis or Warburg effect, is uniquely observed in cancer and the excess generation of lactate that accompanies the Warburg effect needs to be exported from the cell. Elevated EMMPRIN levels have been correlated with invasion and tumor progression in numerous malignant tumor models including melanoma [12], [13]. However, conflicting results regarding the putative involvement of EMMPRIN in the progression of melanoma were reported showing that EMMPRIN expression was observed in non invasive malignant melanoma lesions while both the benign lesions and the most metastatic melanomas were negative [14].

The role of EMMPRIN in tumor progression has been attributed mostly to its protease inducing function. However, Tang et al [15] have recently reported that the up-regulation of EMMPRIN in MDA-MB231 breast tumor cells can also increase VEGF expression in these cells, which can then act in a paracrine manner on endothelial cells to promote tumor angiogenesis. Using two melanoma cell models we show in this study that EMMPRIN can also regulate VEGFR-2 within melanoma cells through HIF-2α, suggesting that EMMPRIN can promote melanoma cell invasion and disease progression by stimulating the VEGF/VEGFR-2 autocrine loop.

## Results

### EMMPRIN up-regulates VEGFR-2 expression in primary melanoma cells

**Figure 1:**
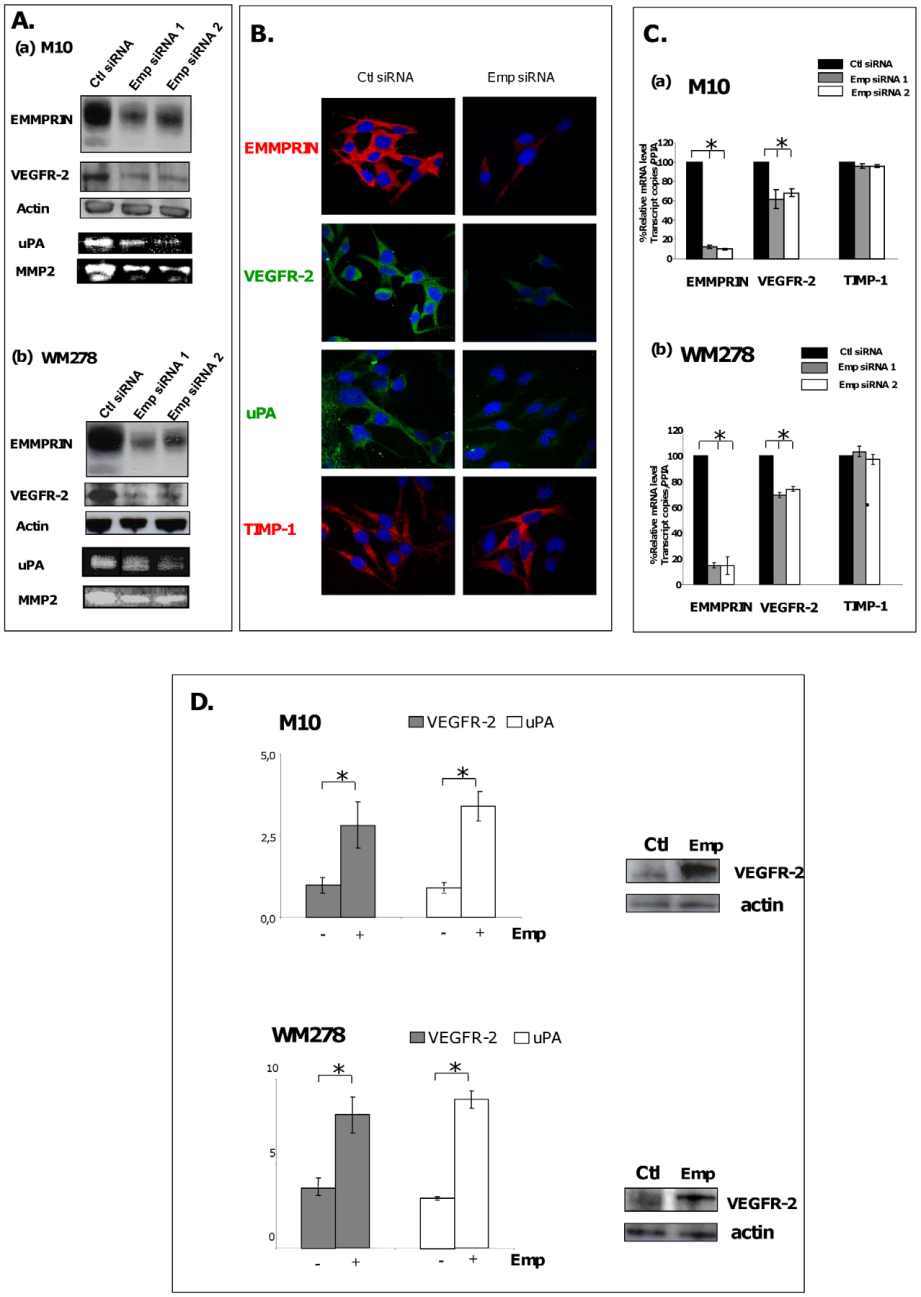
EMMPRIN up-regulates VEGFR-2 expression in primary melanoma cells. **A**. M10 and WM278 cells were transfected for 24 hours with two different EMMPRIN siRNA (Ambion siRNA) or their corresponding scrambled control siRNA (Ctl siRNA) at 33 nmol/L concentration. EMMPRIN and VEGFR-2 protein expression was evaluated by Western blot analysis of 15 µg cell lysates respectively (actin was used as a loading control; representative blot of 3 independent experiments is shown) and conditioned medium (CM) was harvested for gelatinases and uPA zymographies. **B**. Immunofluorescence of M10 cells treated with EMMPRIN siRNA and immunostained for EMMPRIN, VEGFR-2, uPA and TIMP-1. **C**. QRT-PCR analyses of EMMPRIN, VEGFR-2 and TIMP-1 showing the mean ±SD of relative expression to *PPIA* house keeping gene of at least 3 independent experiments. **D**. M10 and WM278 cells were incubated with CHO-membranes containing or not EMMPRIN (Ctl and Emp respectively). VEGFR-2 and uPA transcript levels were quantified by qRT-PCR showing the mean ±SD of relative expression to *PPIA* house keeping gene of at least 3 independent experiments. VEGFR-2 protein expression was evaluated by Western blot analysis of 30 µg cell lysates (actin was used as a loading control; representative blot of 3 independent experiments is shown); bars, SD. *, p<0.05.

The role of EMMPRIN in the regulation of VEGFR-2 production in melanoma cells was investigated by inhibiting its expression using RNA interference strategy. EMMPRIN siRNA transfection of the two melanoma cell lines M10 and WM278 showed a significant reduction in the level of VEGFR-2, as detected by western blot and immunofluorescence analysis (Fig. 1A and B). This reduction was also observed at the transcript level. The average decrease in VEGFR-2 observed using two different EMMPRIN siRNA was 35% in M10 cells and 25% in WM278 cells (Fig. 1C). EMMPRIN down-regulation in melanoma cells was without any effect on VEGFR-1 expression (data not shown). The effectiveness of EMMPRIN siRNA treatment was demonstrated by the reduced EMMPRIN expression in the two cell lines at both mRNA (by 80%) and protein levels (by 50%) and by the resulting inhibition of both MMP-2 and uPA, known to be regulated by EMMPRIN, which were used as positive controls. No detectable effect on TIMP-1 (negative control [16]) expression was observed (Fig. 1).

The regulation of VEGFR-2 by EMMPRIN was also shown by treating the cells with exogenously added EMMPRIN. EMMPRIN contained within membrane vesicles was obtained from CHO cells previously transfected with EMMPRIN full length cDNA. Membrane proteins applied at 25 µg/ml was derived from approximately 25000 CHO-Emp cells. The incubation of M10 and WM278 cells with the EMMPRIN-containing membranes (designated Emp) induced VEGFR-2 (∼2 fold), whereas those prepared from mock-transfected CHO cells (Ctl) had no effect (Fig. 1D). This was accompanied by a 3 fold increase in uPA expression. Thus, endogenous as well exogenously added EMMPRIN regulate VEGFR-2 in two different primary melanoma cell line models, suggesting a more common mechanism not limited to a specific cell line.

### EMMPRIN regulates melanoma cell migration, proliferation and apoptosis through VEGFR-2

**Figure 2:**
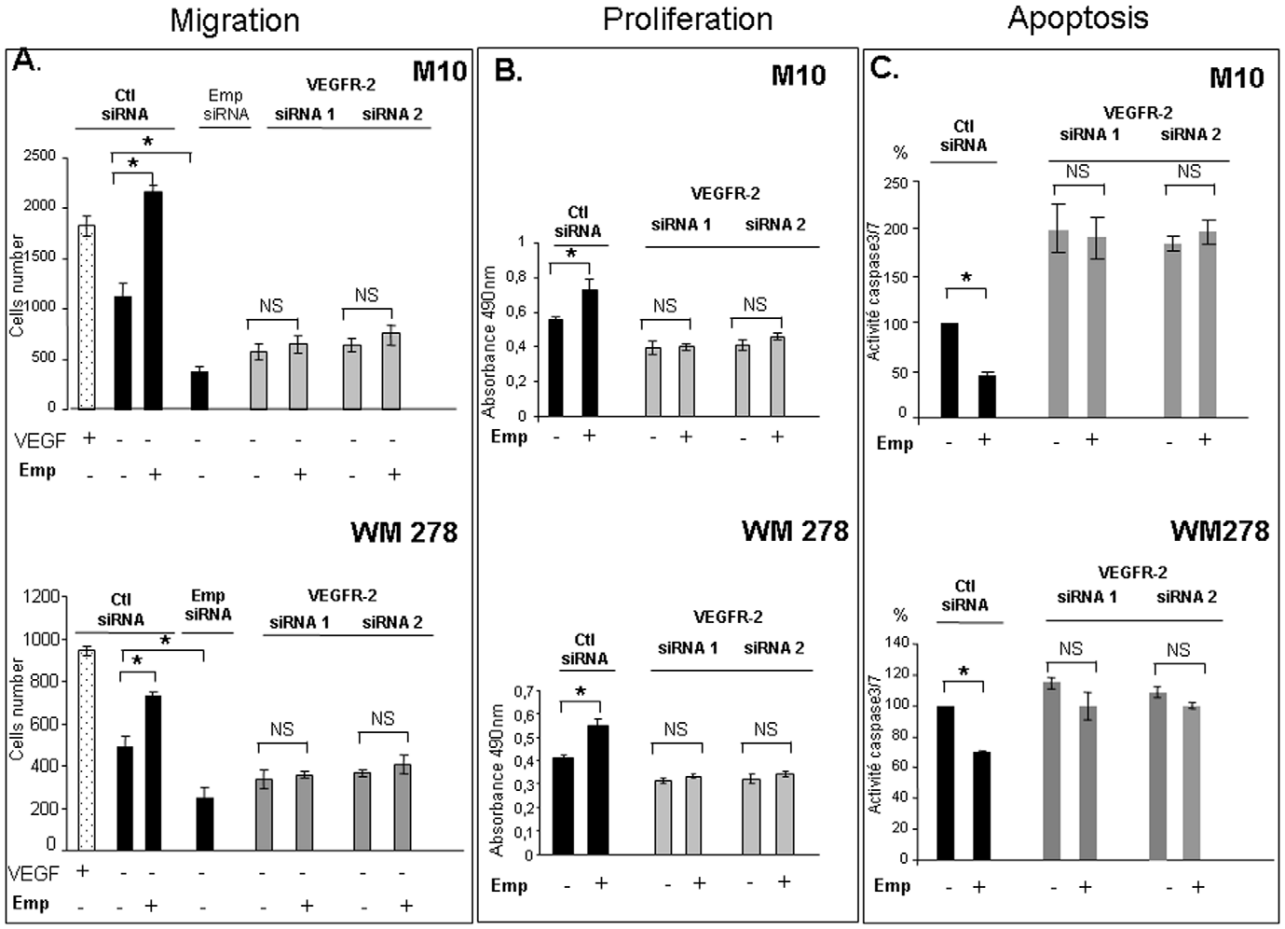
EMMPRIN regulates melanoma cell migration, proliferation and apoptosis through VEGFR-2. **A, Migration**. M10 and WM278 cells seeded in 12-well/insert plates were transfected with EMMPRIN, VEGFR-2 siRNA or scrambled siRNA (Ctl siRNA) before treatment for 24 h in serum-free medium with Emp (25 µg/ml) or with VEGF (200 ng/mL) used as a positive control. After 24 h of incubation, cells were fixed, stained with Diff Quik and counted under a microscope. Columns, means of three independent experiments carried out in triplicate; bars, SD. *, P<0.05. **B, Proliferation**. M10 and WM278 transfected with VEGFR-2 siRNA or scrambled siRNA (Ctl siRNA) were plated onto 96-well plates and treated with Emp (25 µg/ml) for 48 hours in serum-free medium. The bioreduction of tetrazolium (MTS) was performed for 4 h at 37°C, and cell proliferation was quantitatively assessed by measuring absorbance at 490 nm. Results are presented as the mean of three independent experiments carried out in triplicate; bars, SD. *, p<0.05. **C, Caspase-3/7 activity**. Transfected melanoma cell lines with VEGFR-2 siRNA or scrambled control (Ctl siRNA) were seeded onto 96- well plates and treated with Emp (25 µg/ml) for 24 hours in serum-free medium. Apo-one homogeneous Caspase 3/7 buffer containing Z-DEVD-R110 substrate was added to the cells and incubate at room temperature for 4 hours. The caspase-3/7 activity was measured using spectrofluorometer at 530 and 485 nm (excitation and emission wavelength respectively). The data represent means of at least three different experiments; bars, ± SD. * p<0.05.

As VEGF/VEGFR-2 system has been implicated in regulation of melanoma cells migration, proliferation and apoptotic processes, the relationship between EMMPRIN regulation of VEGFR-2 and these cellular properties was investigated. Exogenously added Emp increased cell migration, measured using a modified Boyden chamber assay, showing a 2 fold increase in M10 and a 1.5 fold increase in WM278 compared to control cells. EMMPRIN siRNA reduced cell migration by 66% in M10 and by 50% in WM278 (Fig. 2A). In order to determine if this EMMPRIN's effect on cell migration involves VEGFR-2, the expression of VEGFR-2 was inhibited by siRNA transfection prior to addition of EMMPRIN (Fig. 2A). The VEGFR-2 siRNA transfection inhibited cell migration in both cell lines, although the inhibition was somewhat greater in M10 cells (46%) than in WM278 (28%) (average of two siRNA). However, the addition of EMMPRIN containing membranes to these cells did not restore migration implying that EMMPRIN enhancement of melanoma cell migration requires VEGFR-2.

EMMPRIN also regulated cell proliferation in a VEGFR-2 dependant manner. Exogenously added EMMPRIN caused a 1.4 fold and 1.3 fold increases in cell proliferation in M10 and WM278 respectively, but could not restore the reduced proliferation observed in the VEGFR-2 siRNA inhibited cells (Fig. 2B). Similar results were obtained using PNNAG proliferation assay (data not shown).

We next examined the effect of EMMPRIN on the apoptosis of these melanoma cells. Exogenous EMMPRIN treatment was shown to induce an anti-apoptotic effect in both M10 and WM278 cells compared to control as is shown by the respective 54% and 30% inhibition in caspase 3/7 activity (Fig. 2C). VEGFR-2 siRNA transfection increased the level of caspase3/7 activity by a 2 fold increase (average of two different siRNA) in M10 but weaker increase was observed in WM278 cells (average 12%). However, the addition of EMMPRIN to these VEGFR-2 silenced melanoma cells did not significantly modified caspase 3/7 activity suggesting an anti-apoptotic effect of EMMPRIN through of VEGFR-2. These results were confirmed using annexin-V/7-AAD doubling staining flow cytometry assay (data not shown).

### EMMPRIN up-regulates VEGFR-2 via HIF-2α

**Figure 3:**
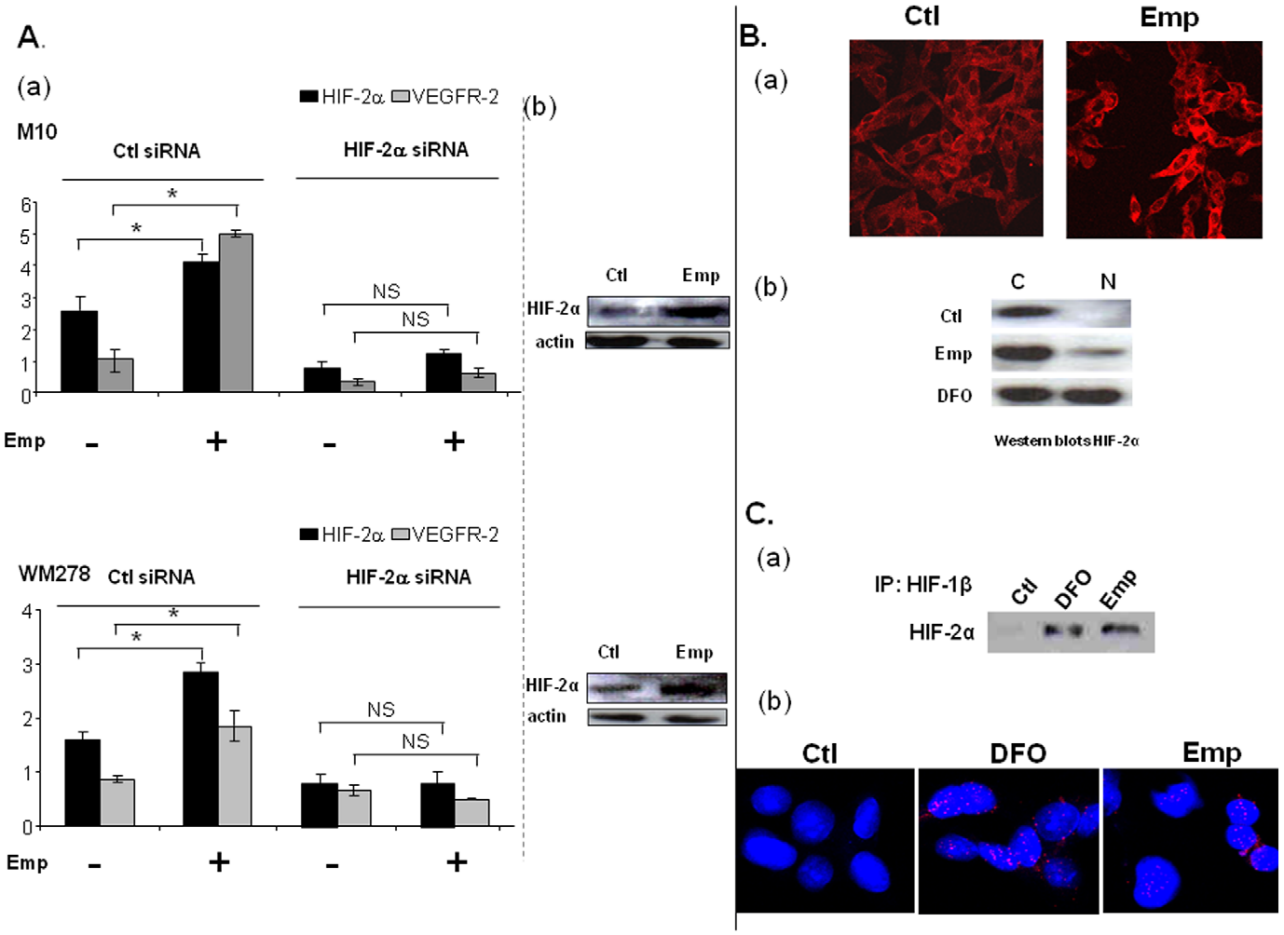
EMMPRIN up-regulates VEGFR-2 through HIF-2α stimulation under normoxic conditions. **A**. Melanoma cells were transfected with HIF-2α siRNA or scrambled siRNA (Ctl siRNA) before treatment with 25 µg/ml of Emp for 1 h. a) HIF-2α and VEGFR-2 transcripts were quantified by qRTPCR (columns, means of relative expression to *PPIA* housekeeping gene of at least three independent experiments; *bars*, SD. * p<0.05). b) HIF-2α protein expression was evaluated by Western blotting. Cells were transfected with scrambled siRNA before Emp treatment. Actin was used as a loading control; representative blot of 3 independent experiments is shown. **B**. HIF-2α nuclear localization after EMMPRIN stimulation of M10 melanoma cells. a) Immunofluoresence analysis of HIF-2α. Cells were grown on the glass slide chamber and treated or not with Emp for 4 h before fixation. HIF-2α protein localization was visualized by confocal microscopy. b) Western blot analysis of the subcellular fractions (cytosoloic (C) and nuclear (N)) of M10 cells treated or not with Emp or 100 µM deferoxamine (DFO), used as positive control, for 4 h before being harvested. C. HIF-2α forms heterodimers with HIF-1β after EMMPRIN stimulation of M10 melanoma cells. a) HIF-2α protein expression was evaluated by Western blotting of the nuclear fraction after immunoprecipitation with HIF-1β antibody. b) In situ PLA detection of HIF-2α and HIF-1β heterodimers: HIF-1β/HIF-2α heterodimers (red) in nuclei of M10 melanoma cells after in situ PLA using antibodies against HIF-1β/HIF-2α. M10 cells were treated or not with Emp or 100 µM deferoxamine (DFO), used as positive control, for 4 hours. Nuclei were stained with DAPI (blue). The EMMPRIN and DFO panels show high magnification to clearly visualize the PLA spots representing heterodimers.

As hypoxia-inducible factor HIF-2α is a key regulator of VEGFR-2 gene expression, we sought to determine whether the increased expression of VEGFR-2 after EMMPRIN treatment may result from a stimulation of this transcription factor. The data in Fig. 3A shows that EMMPRIN treatment up-regulated HIF-2α both at the RNA and protein levels. This increase, already noted after 15 min at the RNA and 30 min at the protein level, preceded that of VEGFR-2 (not shown).

As HIF alpha activity is known to necessitate translocation to the nucleus, we examined the cell distribution of HIF-2α within M10 melanoma cells treated with EMMPRIN by immunofluorescence. As is shown in Fig. 3B, EMMPRIN treated cells exhibited a great increase of HIF-2α staining in the cytoplasm with a number of cells showing its nuclear accumulation, not observed in control cells. The nuclear localization of HIF-2α was also confirmed by western blots of subcellular fractions of M10 cells treated with EMMPRIN similarly to that obtained with 100 µM deferoxamine (DFO), used as a positive control(Fig. 3B).

We next investigated whether HIF-2α forms nuclear heterodimers with HIF-1β after EMMPRIN treatment in M10 melanoma cells. To address that, we used two different approaches: immunoprecipitation and In situ Proximal ligation assay (in situ PLA) using confocal microscopy which highlights protein/protein heterodimers in subcellular compartment. Nuclear extracts immunoprecipitation using HIF-2α antibody followed by western blot analysis with HIF-1β antibody have shown that HIF-1β co-immunoprecipitated with HIF-2α in EMMPRIN and DFO treated cells while it was not detected in control cells. Furthermore, In situ PLA assay in melanoma cells treated for 4 hours with EMMPRIN or DFO (used as positive control) have shown nuclear heterodimers of HIF-2α and HIF-1β not observed in control cells (**Fig. 3C**). These results are in favour of HIF-2α/HIF-1β nuclear heterodimers formation after EMMPRIN treatment.

We next looked at the effect of HIF-2α silencing on EMMPRIN regulation of VEGFR-2. HIF-2α siRNA transfection of both melanoma cell lines down-regulated HIF-2α expression at the mRNA level by ∼67% in M10 and 59% in WM278 cells. This was associated with a decrease in the basal mRNA levels of VEGFR-2 (60% for M10 and 28% for WM278) compared with scrambled control siRNA-transfected cells. The addition of EMMPRIN to the HIF-2α siRNA-treated cells could no longer up-regulate VEGFR-2 (Fig. 3A). These results suggest that HIF-2α plays a central role in EMMPRIN regulation of VEGFR-2 in primary melanoma models.

### EMMPRIN correlation with VEGFR-2 expression and HIF-2α nuclear localization in human melanoma tissues

We next examined human melanoma tissues for possible association between EMMPRIN and VEGFR-2 expression. In a tumor series from 26 melanoma patients, a strong correlation was noted between EMMPRIN and VEGFR-2 transcript levels, measured using qRT-PCR analysis, (Spearman r = 0.82 and r = 0.75 for relative expression to the housekeeping gene transcripts β2 microglobulin and PPIA respectively; both p<0.05). We found a statistically significant correlation between the expression of VEGFR-2 in tumor sample and Clark level (p = 0.44) in this patient population but no significant association between EMMPRIN nor VEGFR-2 and Breslow index, ulceration of the primary tumor and overall survival. However, conducting this analysis on a larger series of patient will be required to establish whether such correlation does exist.

**Figure 4:**
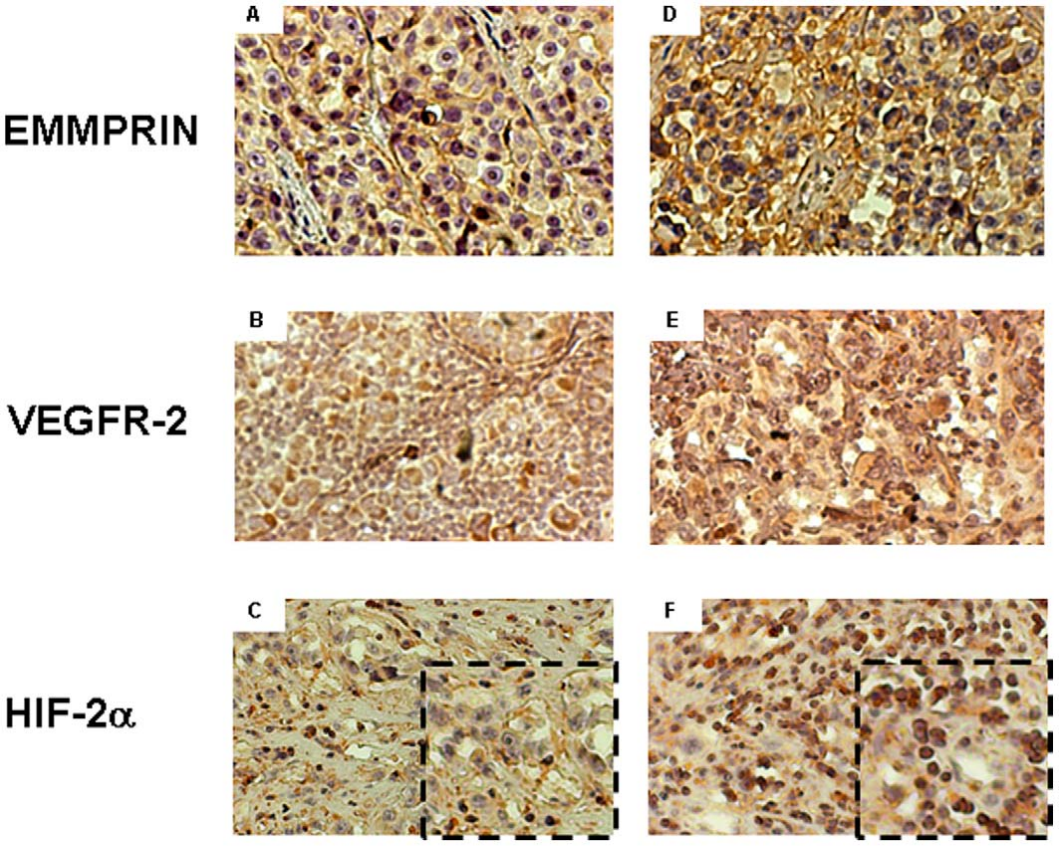
Immunohistochemical staining of EMMPRIN, VEGFR-2 and HIF-2α in sections of human melanoma tissues. **Left**, representative melanoma with lower expression of EMMPRIN. (**A**) EMMPRIN, (**B**) VEGFR-2 and (**C**) HIF-2α staining. **Right**, representative melanoma exhibiting strong expression of EMMPRIN. (**D**) EMMPRIN, (**E**) VEGFR-2, and (**F**) HIF-2α staining.

The expression of EMMPRIN, VEGFR-2 and HIF-2α was also evaluated by immunohistochemical analysis of tumors obtained from 5 of these melanoma patients chosen on the basis of their EMMPRIN expression as determined by the qRT-PCR measurements. Fig. 4 shows a representative staining pattern in tumor tissues of 2 of these patients with low (1 of 2 patients) and high (1 of 3 patients) expression of EMMPRIN. High EMMPRIN levels seemed to be associated with high VEGFR-2 levels in the same tumors. Staining of HIF-2α further shows marked HIF-2α nuclear localization in the higher EMMPRIN expressing tumors, where more than 80% of stained cells in the low EMMPRIN expressing tumors had a non-nuclear localization of HIF-2α. These results support our *in vitro* data and suggest that EMMPRIN may also regulate HIF-2α and VEGFR-2 during tumor progression *in vivo*.

## Discussion

EMMPRIN expression in melanoma tissue correlates with tumor size and staging, and is predictive of poor prognosis [13]. Although EMMPRIN is mainly known for its proteinase inducing ability, it is becoming increasingly clear that it has other functions which may contribute to malignancy. Using two different primary melanoma cell lines we demonstrate in this study that EMMPRIN promotes melanoma cell proliferation and migration and inhibits apoptosis through the specific up-regulation of VEGFR-2. These results which were corroborated by the correlation found between the expression of EMMPRIN and that of VEGFR-2 in human melanoma tissues extend on the proposed protease inducing function of EMMPRIN in tumor invasion and reveal a novel mechanism by which EMMRPIN participates in tumor cell progression.

EMMPRIN has already been shown to regulate VEGF expression in both mammary [15] and melanoma [17], [18] tumor cells, evoking a role in tumor angiogenesis. The secreted VEGF is thought to increase the VEGF pool in the tumor microenvironment and can then act in a paracrine manner on the VEGF receptors on endothelial cells to promote angiogenesis. However, the expression of VEGFR-2 on melanoma cells and its up-regulation by EMMRPIN suggests an autocrine role with consequences on the malignant properties of the tumor cells themselves. Indeed, increasing evidence in recent years support the functional importance of VEGF receptors in cell types other than endothelial cells [19]. Immunohistochemical studies in human melanoma demonstrated that VEGFR-2 expression in the tumor cells was significantly associated with an increased proliferative rate, as Ki-67 was present in 89% of the 202 vertical growth phase melanoma studied [20]. These findings, together with the lack of correlation noted between VEGFR-2 and the microvascular density in these tumors reinforce the idea that the role exerted by VEGF/VEGFR-2 in melanoma is primarily implicated in promoting melanoma cell growth and may be independent of the development of a tumor related capillary network [21], [22]. The autocrine role of VEGFR-2, also supported by *in vitro* studies [23], [24] showing that melanoma cells with higher levels of VEGFR-2 exhibit a greater ability to spontaneously invade the extracellular matrix [21], suggests that EMMPRIN's increase of VEGFR-2 would primarily reflect a role in melanoma cell proliferation and invasion.

Our data also show that EMMPRIN up-regulated VEGFR-2 via HIF-2α, one of the isoforms of the hypoxia inducible factors that mediate adaptation to hypoxic stress in rapidly growing tissues such as tumors. EMMPRIN increased both expression and nuclear localization of HIF-2α. Indeed, immunohistochemical analysis of human melanoma tissues has shown that higher EMMPRIN levels in the tumor cells were associated with a striking increase in HIF-2α nuclear localization. However, in cultured melanoma cells, most of the increased HIF-2α remained cytosolic, suggesting that only a fraction of its nuclear localization is sufficient to stimulate transcription of VEGFR-2. Indeed, this nuclear fraction of HIF-2α was found to form heterodimers with HIF-1β. VEGFR-2 gene was already shown to be regulated in a HIF-2α specific manner as a result of cooperation between the ETS-1 transcription factor and HIF-2α, but not HIF-1α, within the KDR promoter [25]. The fact that, under our experimental conditions, EMMPRIN induction of VEGFR-2 via HIF-2α occurs under normoxic conditions is in accord with the demonstrated higher stability of HIF-2α than HIF-1α at higher oxygen tensions. This stability was attributed to the cytoplasmic trapping of HIF-2α which was suggested to protect it from oxygen-dependent protein degradation under normoxia, and its overexpression and accumulation in the cytoplasm was shown to induce its translocation to the nucleus to stimulate gene induction [26]. The higher stability of HIF-2α is thought to contribute to the development of tumor aggressiveness by inducing the program for a hypoxic phenotype even at near physiological oxygen tensions [27]. We suggest therefore that by inducing HIF-2α under normal oxygen levels, EMMRPIN may contribute to the acquisition of a phenotype with aerobic glycolysis, referred to as the “Warburg phenomenon”, leading to lactate production and acid microenvironment and the evolution of acid- resistant cell phenotypes with greater tumor aggressiveness [28]. EMMPRIN was already shown to increase lactate release and uptake, thus enhancing glycolytic flux only in cancer cells including melanoma but not in normal fibroblasts [29].

Little is known at present on the mechanisms by which EMMPRIN regulates the production of MMPs or its other targets such as uPA or VEGF. It is conceivable that a common pathway exists for EMMPRIN regulation of both VEGFR-2 and the matrix degrading proteases and which would recapitulate clinical findings in which MMP and uPA expression in tumor tissues often correlates with that of the VEGF/VEGFR system and tumor invasiveness. On the basis of our findings that the regulation of VEGFR-2 by EMMRPIN in melanoma cells is mediated through HIF-2α, results strengthened by their correlation in human melanoma tissues, it is tempting to speculate that HIF-2α represents a central pathway for the increased expression of these EMMPRIN target genes. Indeed, elevated HIF-2α levels have already been associated with MT1-MMP, another EMMPRIN target gene, as well as VEGF and VEGFR-2 [25].

The implication of VEGFR-2 in tumor progression suggests that its inhibition could be beneficial in the clinical context. Indeed, the use of VEGFR-2 blocking antibody in experimental melanoma models significantly inhibited tumor growth [30]. The inhibition of EMMPRIN in melanoma can represent an alternative therapeutic strategy as it is expected to inhibit cell growth and invasion through the simultaneous inhibition of the VEGFR-2/VEGF system and the matrix degrading proteases and possibly other tumor promoting HIF-2α targets.

## Materials and Methods

### Cell Culture

Primary melanoma cell lines WM278 (Wistar Institute) and M10, both established from patients primary nodular melanoma, were cultured as previously described. WM278 cell line is maintained in a 4∶1 mixture of MCDB153 (VWR, France) and Leibovitz's L-15 (Invitrogen, France), supplemented with 2% Fetal bovine serum (FBS) and 5 µg/ml insulin (Sigma, France). M10 cells were maintained in RPMI-medium supplemented with 10% FBS, Hepes 1M, Pyruvate Na, and L-Glutamine (Invitrogen) [31].

Chinese Hamster Ovary (CHO) cells (ATCC, Rockville, MB) were cultured in DMEM/F12 (Invitrogen) supplemented with 10% FBS and 2 mML-glutamine. CHO cells were transfected with a plasmid containing or not EMMPRIN full-lengh cDNA as previously described [21] and stably transfected cells were designated as CHO-Emp cells and CHO-Mock cells. CHO-Emp and CHO-Mock membranes were isolated by differential centrifugation as previously described [32]. The bioactivity of EMMPRIN-containing membranes was verified by its activity in stimulating uPA expression in melanoma cells [10]. The membrane vesicles obtained from the CHO-Emp or CHO-Mock cells will be referred to as Emp or Ctl respectively throughout.

### Small interfering RNA transfection

siRNA for VEGFR-2 (IDs: 220 and 221), HIF-2α (IDs: s4700 and s4699) and EMMPRIN (IDs: 147251 and 215973) or scrambled siRNA oligos (Ambion/Applied-Biosystems, France) were transfected into cells by using the Block-iT transfection kit and Lipofectamine-2000 (Invitrogen). Cells were then incubated for 24 h before analysis for qRT-PCR, Western Blotting, migration, apoptosis and proliferation and for a further 24 hours in serum-free medium for zymographic analysis.

### Western Blotting analysis

Western Blots were preformed as described previously [32]. Membranes were immunoblotted with anti-EMMPRIN (HIM6, BD, Pharmingen), anti-HIF-2α (ep190b, Novus-Biologicals) or anti-VEGFR-2 (Sc-504, Santa Cruz-biotechnology) antibodies. Proteins were visualized with ECL reagent (Pierce) and their relative expression was determined by densitometry using ImageJ software program and normalized relative to β-actin.

### RNA extraction, reverse transcription and Real-Time quantitative PCR (qRT-PCR)

Total RNA was isolated using Trizol (Invitrogen). cDNA was synthesized using random hexamers and M-MLV (Invitrogen). EMMPRIN, VEGFR-2, HIF-2α and TIMP-1 mRNA levels were measured by qRT-PCR using Perfect MasterMix-Probe (AnyGenes, France) on LightCycler 2.0 (Roche) according to the manufacturer's protocols. The expression levels of interest transcripts were normalized to the housekeeping PPIA (peptidylprolyl isomerase A) transcripts. Primers and probes (Eurogentec, Belgium) are available upon request.

### Zymographic analysis

Gelatin or casein zymography was performed as described previously [10]. Serum-free conditioned media were analyzed on 10% SDS-PAGE gels containing either 1 mg/mL gelatin for gelatinase activity or 2 mg/mL casein (Sigma) and 10 µg/mL plasminogen (Calbiochem) for uPA activity.

### Immunofluorescence, confocal microscopy

Cells previously transfected with EMMPRIN-siRNA were fixed and incubated with primary anti-VEGFR-2/KDR (Sc-504, Santa Cruz Biotechnology), anti-EMMPRIN (HIM6, BD-Pharmingen), anti-uPA (American Diagnostica) and anti-TIMP-1 (Calbiochem) antibodies. In other experiments cells were treated or not with Emp (defined in Cell Culture section) were fixed and incubated with anti-HIF-2α (ep190b, Novus-Biologicals) antibody. After incubation with the corresponding secondary antibody (Alexa 488 and 594) the slides were examined with a laser-scanning confocal microscope (Leica Lasertechnik, Heidelberg). In the negative controls the primary antibody was substituted with PBS.

### In situ proximity ligation assay (in situ PLA)

Cells treated with 25 ng/mL Emmprin or 100 µM deferoxamine (DFO) for 4 hours were immediately fixed; thereafter subjected to in situ PLA using Duolink Detection kit (Olink Bioscience, Uppsala, Sweden) according to the manufacturer's instructions for Duolink Blocking solution and Detection protocol. Briefly, slides were blocked, incubated with antibodies directed against HIF-2α (1∶100; ep190b, Novus-Biologicals) and HIF-1α (1∶150; Santa cruz biotechnology) and thereafter incubated with PLA probes, which are secondary antibodies (anti-mouse and anti-rabbit) conjugated to unique oligonucleotides. Circularization and ligation of the oligonucleotides was followed by an amplification step. The products were detected by a complementary fluorescently labelled probe. The heterodimers visualized as bright fluorescent signals were examined with a laser-scanning confocal microscope (Leica Lasertechnik, Heidelberg). Representative results are shown from experiments repeated at least three times.

### Subcellular protein extraction and fractioning

In order to analyze HIF-2α nuclear localization, cytosolic and nuclear fractions were isolated from 80% confluent cells by using ProteoExtract Subcellular Proteome Extraction Kit (Calbiochem), based on differential centrifugation in density gradients. The cytosolic and nuclear fractions were separated by SDS-PAGE and analyzed by Western blotting with a mouse anti-HIF-2α antibody.

### Co-immunoprecipitation assay

Cultures grown in 100 mm dishes (10^6^/dish), treated or not with Emp or DFO, were lysed. The protein lysate was processed for immunoprecipitation with 1 µg of HIF-1β antibody (Santa cruz biotechnology). Immunoprecipitates were collected using Protein G Sepharose (Sigma) and subjected to SDS–PAGE, followed by transfer to a nitrocellulose membrane and probed with antibody for the detection of HIF-2α. Assays were repeated three times and representative results are shown.

### Migration assay

The *in vitro* migration assay was performed using a modified Boyden chamber [33]. Emp (25 µg/mL) or VEGF (200 ng/mL positive control) (R&D-Systems) was added to the cells previously transfected with VEGFR-2-siRNA and seeded on the upper chamber of the insert. After 24 h of incubation, cells were fixed, stained with Diff-Quik (Dade Behring, Deerfield, IL), and counted under a light microscope.

### Cell apoptosis and proliferation assays

Cells seeded into 96-well plates and transfected with VEGFR-2 siRNA were treated with 25 µg/ml of Emp prior to active caspase 3/7 detection using the Apo-ONE Homogeneous Caspase-3/7 Assay kit (Promega) according to the manufacturer's protocol. Apoptosis was also assessed by measuring membrane redistribution of phosphatidilserine using an annexin V-FITC apoptosis detection kit.

Proliferation of WM278 and M10 was measured using the CellTiter 96 Aqueous Non Radioactive Cell Proliferation Assay (Promega).

### Analysis of human melanoma tissues

Tumor samples were collected from 26 patients with melanoma with their informed consent. Frozen and formalin-fixed and paraffin-embedded tissue sections were analysed by both qRT-PCR and immunohistochemistry respectively. The study was performed in accordance with the precepts established by the Helsinki Declaration and approved by the Hopital Saint Louis Research Ethic Committee (Paris, France). All patients gave a written consent. All data were analyzed anonymously. Total RNA was extracted from frozen tissue sections using TRIzol reagent (Invitrogen). First-strand cDNA was synthesized using the High-Capacity cDNA Archive Kit (Applied-Biosystems). Transcript levels were measured in each melanoma tissue by qPCR using Perfect Master Mix-Probe (AnyGenes, France) on LightCycler 2.0 System (Roche). The expression levels of interesting transcripts were normalized to the housekeeping β2 microglobulin (B2M) and PPIA gene transcripts. All experiments were performed in duplicates and Spearman's rank correlation was used to evaluate the association between mRNA levels in melanoma tissues. Paraffin-embedded sections were stained with anti-EMMPRIN (HIM6, BD-Pharmingen), anti-VEGFR-2/KDR (MAB3572, R&D-Systems, Minneapolis, MN) or anti-HIF-2α (ep190b, Novus-Biologicals) antibodies. Staining was visualized using the Vectastain Elite universal ABC-kit (Vector Laboratories, Burlingame, CA), followed by counterstaining with Mayer's hematoxylin. Three-amino-9-ethyl-carbazole (Sigma-Aldrich, France) was used as the chromogen.

### Statistical analysis

Data are presented as the mean values ± SD. Mann-Whitney test was used to evaluate differences between groups. P<0.05 was accepted as significant. Pairwise associations between the different markers were assessed by the use of Spearman rank correlation coefficient. The association between markers and clinical parameters was tested by the use ofWilcoxon rank sum test. The prognostic value of the studied biomarkers was evaluated for relapse free survival (RFS) and for overall survival (OS). All tests were 2-sided at a P<0.05 significance level. Analyses were performed by the use of statistical software (R version 2.4.0; R Foundation for Statistical Computing, Vienna, Austria).
